# Skin Grafting

**Published:** 1888-12

**Authors:** Richard J. Levis


### Skin Grafting.

To the Editor.

*Sir:* To an observer of the literature of the progress of practical surgery it is curious to see how frequently old, discarded practices are revived and proclaimed as novelties. Your recent editorial on "Transplantation of Skin From a Corpse" is a marked illustration of such a useless resurrection of a long defunct subject.

Many years ago, soon after the success of the process of skin grafting was announced, I tried grafting from the integument of recently amputated limbs, and also from the cadaver. The success of these grafts was quite equal to that from the living subject. But I soon discontinued the practice of grafting from the cadaver and from amputated limbs, and from any source but the patients' own integument, and publicly deprecated and condemned it as subjecting the patient to serious risks of specific infection.

You need not be troubled by the fear that the recent revival of this improper operation by a German surgeon "is likely to imperil the claim of our country for having originated it." I believe that I was the first who experimentally performed it, but would prefer to make the claim of having been the first to discard and condemn it.

I have successfully transplanted the skin of a rabbit to a raw surface that was denuded in a plastic operation on the face. In a noted case of cicatricial deformity of the face I successfully formed an entire upper eyelid from the integument of an anchylosed and useless finger that I had previously amputated from the patient's own hand.

I send to you herewith a copy of my pamphlet on skin grafting, which was originally published in the *Philadelphia Medical Times* about twelve years ago.

Yours truly,

Richard J. Levis, M. D.

Tobacco on the Eyes.—The eyes of some are especially susceptible to tobacco. Those who feel the slightest nausea or giddiness after using tobacco should let it alone.

